# Surgical Techniques, Outcomes, Indications, and Complications of Simultaneous High Tibial Osteotomy and Anterior Cruciate Ligament Revision Surgery: A Systematic Review

**DOI:** 10.1007/s11420-018-9630-8

**Published:** 2018-09-24

**Authors:** Arnav Gupta, Tushar Tejpal, Ajaykumar Shanmugaraj, Nolan S. Horner, Nicole Simunovic, Andrew Duong, Olufemi R. Ayeni

**Affiliations:** 1 Faculty of Health Sciences, McMaster University, Hamilton, Ontario Canada; 2 Department of Surgery, Division of Orthopaedic Surgery, McMaster University Medical Centre, McMaster University, 1200 Main St West, 4E15, Hamilton, Ontario L8N 3Z5 Canada; 3 Department of Health Research Methods, Evidence, and Impact, McMaster University, Hamilton, Ontario Canada

**Keywords:** high tibial osteotomy, anterior cruciate ligament, posterior tibial slope, knee

## Abstract

**Background:**

The incidence of primary anterior cruciate ligament reconstruction (ACLR) failure ranges from 10 to 20% in the USA. Many patient and surgical factors may lead to re-rupture after ACLR. Some authors have suggested that not correcting excessive posterior tibial slope may be a significant contributing factor to ACLR failure.

**Purposes:**

We sought to systematically review the literature on outcomes, indications, and complications in patients undergoing simultaneous high tibial osteotomy (HTO) and ACLR revision.

**Methods:**

PubMed, Medline, and Embase were searched in February 2018 for articles addressing simultaneous HTO and ACLR revision in skeletally mature patients. Major orthopedic conferences were screened in duplicate to find gray literature. All studies were assessed using the Methodological Index for Non-Randomized Studies. Descriptive statistics are presented where applicable.

**Results:**

Seven studies satisfied inclusion. Seventy-seven patients underwent combined HTO and ACLR revision. The main indications were a posterior slope of more than 12° or severe varus malalignment. Graft types included hamstring tendon autograft (58.4%; *n =* 45) and quadriceps tendon graft (16.9%; *n =* 13). Mean delay between primary and revision surgery was 9 years. Rehabilitation protocol dictated return to running at 4 months and return to sport at 4 to 9 months. Visual analog scale pain scores reduced on average by 30 points. Subjective International Knee Documentation Committee, Tegner–Lysholm, and Tegner activity scores also improved. Fifty-eight percent (35/60) of patients showed osteoarthritis signs post-operatively. One patient (1.3%) received an arthroscopic arthrolysis of adhesions for stiffness. There were no reported incidences of graft re-rupture.

**Conclusion:**

This systematic review found that the use of HTO for ACLR revision produces good post-operative functional outcomes, low complication rates, and no reported re-ruptures. The main indications for combined HTO with ACLR revision was a posterior slope of more than 12° or severe varus malalignment. Future studies with large sample sizes and long-term follow-up are required to corroborate these results.

**Electronic supplementary material:**

The online version of this article (10.1007/s11420-018-9630-8) contains supplementary material, which is available to authorized users.

## Introduction

Although primary anterior cruciate ligament reconstruction (ACLR) surgeries typically have high success rates, there remains the possibility of graft re-rupture mandating revision surgery [27]. The incidence of primary ACLR failure ranges from 10 to 20% in the USA [5]. Unfortunately, revision surgeries produce inferior patient outcomes than primary ACLR [25, 26].

Many patient and surgical factors likely lead to re-rupture after ACLR, including not correcting excessive posterior tibial slope [3, 14, 16, 21, 24]. An excessive posterior tibial slope has previously been defined as anything greater than 12° [5]. It results in anterior shift of the tibia’s resting position, thus increasing anterior translational forces on the ACL. Furthermore, sagittal imbalances of the tibia can lead to abnormal loading of a knee compartment, resulting in damage to menisci and articular cartilage [22].

High tibial osteotomy (HTO) provides promise in correcting pathological posterior tibial slope and varus malalignment by re-aligning the proximal tibia’s bony morphology. Studies have found that HTO correction of a pathological posterior tibial slope can reduce anterior laxity in patients with ligamentous instability [2, 4, 20]. Likewise, reduced posterior tibial slopes may actually have a protective effect on the ACL graft, thereby reducing the re-rupture rate [23]. Furthermore, while ACLR can improve knee biomechanics, HTO can delay the progression of osteoarthritis by controlling anterior tibial translation and offloading the medial compartment of the knee.

Many surgeons may be hesitant to routinely perform HTO in primary ACLR patients, due to its difficulty and the added risk of associated complications, especially since the success rate of primary ACLR without HTO is quite high [7, 10]. However, some surgeons may argue that a combined HTO–ACLR procedure can improve outcomes and is appropriate in the setting of ACLR revision and pathologic posterior tibial slopes or varus deformity.

Based on previous systematic reviews, it can be hypothesized that reducing posterior tibial slope to so-called non-pathological levels (i.e., less than 12°) may be beneficial for ACLR revision patients. However, to date, no systematic reviews have critically evaluated the impact of simultaneous HTO in ACLR revision. Hence, we sought to systematically assess the outcomes, indications, and complications in patients undergoing simultaneous HTO and ACLR revision.

## Methods

The Preferred Reporting Items for Systematic Reviews and Meta-analyses (PRISMA) guidelines were followed for this review [12].

### Design and Eligibility Criteria

The research question and study eligibility criteria for this systematic review were established a priori. The inclusion criteria were studies involving (1) levels I to IV evidence, (2) skeletally mature patients (i.e., closed femoral and tibial physes) at the time of HTO and ACLR revision, and (3) outcomes of simultaneous HTO and ACLR revision. The exclusion criteria were studies involving (1) primary ACLR, (2) skeletally immature patients, (3) non-surgical treatment (e.g., articles on conservative treatment), (4) cadavers or non-human subjects, and (5) reviews.

### Search Strategy

One reviewer (AS) searched three online databases (Embase, Medline, and PubMed) for studies related to HTO and ACLR revision from database inception to February 27, 2018. The following key terms were used in a broad-based search: “anterior cruciate ligament,” “osteotomy,” and “slope” and is outlined in detail in Appendix. The search terms were entered into Google Scholar, a search engine for scholarly literature from various disciplines and sources (e.g., articles, theses, and books), to ensure that articles were not missed. References of included studies were also screened using the same systematic approach to capture additional relevant articles.

### Study Screening

Two independent reviewers (AG and TT) screened titles, abstracts, and full text of the retrieved studies in duplicate. Discrepancies during title and abstract screenings were resolved by automatic inclusion to ensure no relevant articles were missed during screening. Discrepancies during full-text screening were resolved through consensus between the reviewers. If a consensus could not be reached, input from a third senior reviewer (AS) resolved the discrepancy. A manual search through Google Scholar and included full-text references was conducted to ensure no relevant papers were missed. The following orthopedic conferences were screened in duplicate for gray literature (any literature not found through commercial publishers): American Association of Hip and Knee Surgeons (the 24th through 27th annual meetings), International Society of Arthroscopy, Knee Surgery and Orthopaedic Sports Medicine (10th and 11th biennial congresses, 2015 and 2017), Vail Hip Symposium (2015 through 2017), American Orthopaedic Society for Sports Medicine (2014 through 2017), and American Academy of Orthopaedic Surgeons (2014, 2015).

### Quality Assessment of Included Studies

Using the *Journal of Bone and Joint Surgery* classification system for literature in orthopedics, the level of evidence (I to IV) for each study was determined by two reviewers (AG and TT) independently and in duplicate [27]. The methodological quality of included studies was assessed using the Methodological Index for Non-Randomized Studies (MINORS) appraisal tool. MINORS is a validated scoring tool for non-randomized studies (e.g., case reports, case series, and cohort studies) [17]. A score of 0, 1, or 2 is given for each of the 12 items on the MINORS checklist, with a maximum score of 16 or 24 for non-comparative or comparative scores, respectively. The two reviewers discussed any disagreements with a senior author until they reached consensus. Methodologic quality was categorized a priori as follows: 0 to 6 to indicate very low-quality evidence, 7 to 10 to indicate low-quality evidence, 10 to 14 to indicate fair-quality evidence, and over 16 to indicate good-quality evidence for non-randomized studies.

### Data Abstraction

Two reviewers (AG and TT) independently abstracted relevant data from included articles. The data was recorded in a Microsoft Excel spreadsheet designed a priori. Information recorded included the author, year of publication, study design, study location, level of evidence, patient demographics, and mean follow-up. Additionally, data on the surgical techniques, rehabilitation protocols, and radiographic and clinical outcomes was also recorded. If studies failed to separate data by surgery type (i.e., primary versus revision ACLR), authors were contacted via email to retrieve the data pertaining to revision patients.

### Statistical Analysis

At the end of each screening stage, a *κ* value was calculated to evaluate inter-reviewer agreement. The agreement was categorized a priori as follows: a *κ* value greater than 0.6 indicated substantial agreement; a *κ* value between 0.2 and 0.6 indicated moderate agreement; and a *κ* value less than 0.21 showed slight agreement [8]. An intra-class correlation coefficient (ICC) was used to evaluate the inter-reviewer agreement of the MINORS score. Descriptive statistics such as means, mean difference, and measures of variance (e.g., standard deviation, 95% confidence interval [CI]) are presented where applicable. A meta-analysis was not conducted due to variability in patient demographic data.

## Results

### Study Characteristics

**Fig. 1:**
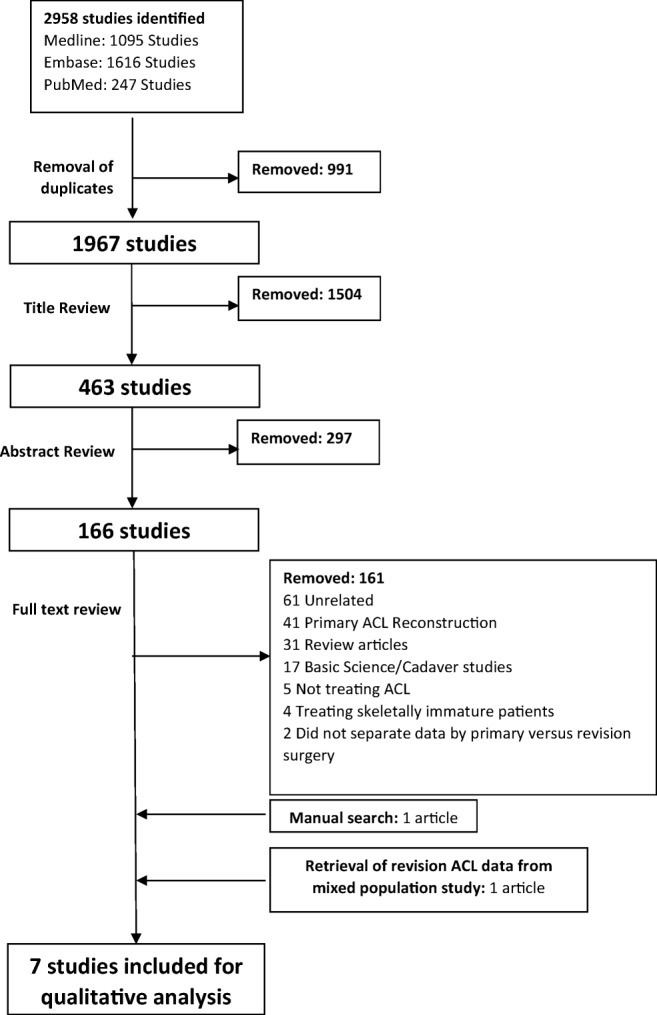
Screening process using the Preferred Reporting Items for Systematic Reviews and Meta-analyses (PRISMA) guidelines. *ACL* anterior cruciate ligament.

The initial search from all databases yielded a total of 2958 articles. After excluding 991 duplicates, a systematic screening process yielded five articles that met inclusion. Missing data pertaining to revision patients in a mixed-population (primary and revision) case series from one study was obtained by contacting the corresponding author [30]. Moreover, upon reviewing references of included studies and a search on Google Scholar, an additional article was retrieved, yielding a total of seven articles (Fig. 1). There were no additional studies found upon reviewing abstracts presented at major orthopedic conferences. Reviewers agreed substantially at screening stages of title (*κ* = 0.89; 95% CI, 0.86 to 0.91) and abstract (*κ* = 0.85; 95% CI, 0.80 to 0.90) and agreed almost perfectly at full-text screening (*κ* = 0.94; 95% CI, 0.87 to 1.00). The seven studies included one prospective cohort, two retrospective cohorts, two case series, and two case reports and were conducted in France (four studies), Italy (two), and Japan (one) (Table 1).

**Table 1:** Study characteristics and methodological quality

Author	Year	Location	Study design (level of evidence)	Sample Size	% male	Mean age (years)	Mean follow-up (months)	Consensus MINORS score*
Baverel et al. [1]	2015	France	Retrospective cohort (III)	11	NR	36 (26–42)	78	10
Dejour et al. [5]	2015	France	Retrospective cohort (III)	10	60	30.3 ± 4.4	48 ± 24 (median 3.6; range 2.0–7.6)	10
Sonnery-Cottet et al. [19]	2014	France	Case series (IV)	5	80	24 (range, 16–40 )	31.6 (range, 23–45 )	11
Walker et al. [23]	2015	USA	Case report (IV)	1	100	22	24	10
Yonekura et al. [29]	2018	Japan	Case report (IV)	1	100	49	3	10
Zaffagnini et al. [30]	2012	Italy	Case series (IV)	13	100	37.4	78 ± 32.4	10
Zaffagnini et al. [31]	2013	Italy	Prospective cohort (III)	36	NR	40 ± 8.1	N/A	9

*NR* not reported, *MINORS* Methodological Index for Non-Randomized Studies

*Through compiled MINORS rating, non-comparative studies were scored from 0 to 16 while comparative studies were scored from 0 to 24

### Patient Characteristics

The included studies had a total of 77 patients. Among the treated population, 83.3% (25/30) were male (two studies did not specify sex distribution [1, 31]). Patients’ mean age was 37.7 ± 6.9 (range, 16 to 54) years, with a follow-up time of 71.5 ± 31.3 (range, 12 to 192) months. At final follow-up, 71 patients were available (92.7%). The mean delay between primary and revision surgery was noted in three studies, with a mean time of 9.0 ± 2.9 years [5, 30, 31]. One study provided age at index ACLR of a patient who was 16 years old and in whom HTO and ACLR were performed 3 years later [19].

### Study Quality

The mean MINORS score for included studies was 9.8 *±* 0.8 (range, 9 to 18), indicating a low quality of evidence for non-randomized studies. All studies were of level III (*n =* 3) or level IV (*n =* 4) evidence (Table 1). There was agreement among reviewers’ MINORS scores (ICC = 0.9; 95% CI, 0.3 to 1.0).

### Indications

All studies discussed indications for performing ACLR with HTO. In four studies, surgeons performed ACLR and HTO in patients with posterior tibial slope of greater than 12°. In two studies, surgeons performed ACLR and HTO in patients with severe varus alignment. Severe varus alignment was considered a hip-knee-ankle angle of greater than 180° [30]. In three studies, surgeons performed the procedure in patients with one previously failed ACLR (*n =* 50) [29–31]; in three studies, they performed the procedure in patients with two previously failed ACLRs (*n =* 26) [1, 5, 19]; and in one study, they performed the procedure in patients with three previously failed ACLRs (*n =* 1) [23].

### Surgical Techniques

In all studies in this systematic review, surgeons performed ACLR with a valgus-producing HTO. Two studies (*n =* 4) utilized a medial opening-wedge approach [7, 29], whereas five studies (*n =* 56) used a lateral closing-wedge approach [5, 19, 23, 30, 31].

Prior to surgery, five patients underwent meniscal repair procedures [5, 23]. In eight patients, a notchplasty was concurrently performed [5, 19]. Thirty-six patients were treated with ACLR along with extra-articular lateral tenodesis [31].

The graft choices included hamstring tendon autografts used in 45 patients (58%) [5, 31], quadriceps tendon autograft used in 13 patients (16.9%) [5], patellar tendon graft used in four patients (5.2%) [23, 30], and Achilles allograft used in four patients (5.2%) [30]. The graft type was unspecified in one study (14.3%; *n =* 11) [1].

The ACL fixation method varied. In two studies, screws were used [5, 19]; in one study, the hamstring distal graft was fixed with one interference screw in both femoral and tibial tunnels [5]; and in another study, the bioabsorbable interference screw only was used at the tibial tunnel [19]. One study used two staples for ACL fixation at the tibial tunnel [30].

HTO fixation technique also varied. In one study, medial and lateral 2.7-mm locking plates were used [23], whereas in another study, double spike plates, along with the TomoFix^™^ Medial High Tibial standard plate, were used [29]. In one study, the osteotomy was fixed using two staples inserted on either side of the patellar tendon [5]. Two studies did not specify the type of fixation used [1, 31].

### Rehabilitation Protocol

The rehabilitation protocol was reported in six of the included studies (*n =* 67 patients). In all studies, non-weight-bearing periods ranged from 3 to 8 weeks post-operatively, followed by progressive non-aggressive rehabilitation with passive and active extension exercises based on patient tolerance [5, 19, 29–31]. Return-to-sports times differed in the included studies and were permitted between 4 and 9 months post-surgery [5, 19, 23, 30].

### Clinical Objective Outcomes

#### Tibial Anterior Translation

Tibial anterior translation was measured in two studies (*n =* 11) (one study used a KT-2000 arthrometer, and one study used a TELOS Stress Device with 150N at 20° knee flexion) [5, 29]. Mean anterior tibial translation decreased from 11.8 mm (95% CI, 11.6 to 12.1) pre-operatively to 4.8 mm (95% CI, 3.8 to 5.8) post-operatively (Table 2).

**Table 2:** Clinical objective outcomes

Author	Tibial anterior translation	Anterior laxity	Posterior tibial slope	Objective IKDC	Osteoarthritis
Dejour et al. [5]	11.7 ± 5.2 mm (pre-op) to 4.3 ± 2.5 mm (post-op)	9 negative pivot shift 1 grade 1	13.2° ± 2.6° (pre-op) to 4.4° ± 2.4° (post-op)	4C, 5D (pre-op) to 7B, 2C (post-op)	2 patients demonstrated development (1 from stage 0 to stage 1, another from stage 0 to stage 2)
Zaffagnini et al. (2013) [31]	NR	2.710 mm at follow-up (3 patients > 5mm)	Decreased by 1.2°	1B, 13C, 18D (pre-op) to 15A, 14B, 3D (post-op)	Significant change in medial compartment only (*p* = 0.023)
					1A, 12B, 19C (pre-op) to 10B, 14C, 8D (post-op)
Zaffagnini et al. (2012) [30]	NR	NR	NR	NR	WOMAC 61.10 ± 11.30 (post-op) to 75.19 ± 7.80 (follow-up)
Baverel et al. [1]	NR	NR	NR	NR	NR
Sonnery-Cottet et al. [19]	NR	10.4 mm (pre-op) to 2.8 mm (post-op)	13.6 (pre-op) and 9.2 (post-op)	3C, 2D (pre-op) to 1A, 4B (post-op)	NR
		4 negative pivot shift, 1 grade 1			
Walker et al. [23]	N.R	1 negative pivot shift	NR	NR	NR
Yonekura et al. [29]	13 mm (pre-op) to 10 mm (post-op)	1 negative pivot shift	17° (pre-op) to 15° (post-op)	NR	No OA signs; JOA score improved from 85 points to 100 points

*NR* not reported, *IKDC* International Knee Documentation Committee, *JOA* Japanese Orthopaedic Association, *OA* osteoarthritis, *WOMAC* Western Ontario and McMaster Universities Arthritis Index

#### Anterior Laxity

Anterior laxity was measured in five studies (*n =* 53) [5, 19, 23, 29, 31]. Mean anterior laxity, as measured by side-to-side difference, reduced from 10.4 mm (95% CI, 10.4 to 10.4; *n =* 5) pre-operatively to 2.7 mm (95% CI, 2.7 to 2.7; *n =* 41) post-operatively (one study reported *p* < 0.01, and in one study, the *p* value was not reported [NR]), of which 7.3% (*n =* 3) of patients had more than 5-mm side-to-side difference. Pivot shift test results were reported in four studies (*n =* 17): 88.2% of patients had a negative pivot shift post-operatively (Table 2) (in four studies, *p* value was NR).

#### Posterior Tibial Slope

Reductions in posterior tibial slope were reported in four studies (*n =* 52) [5, 19, 29, 31]. The mean reduction in posterior tibial slope was 3° (95% CI, 2.2 to 3.8) (one study reported *p* < 0.01; in three studies, *p* value was NR). Of the four studies reporting posterior tibial slope, three studies (*n =* 16) reported mean posterior tibial slopes that were greater than 12° pre-operatively [5, 19, 29]. The mean reduction in posterior tibial slope was 7° (95% CI, 5.9 to 8.1) (one study reported *p* < 0.01; in two studies, *p* value was NR). Only one study (*n =* 36) reporting posterior tibial slope concurrently indicated varus alignment [31]. The mean reduction in posterior tibial slope was 1.2° (*p* value was NR). Three studies (*n =* 51) reporting posterior tibial slope indicated the use of a closing-wedge osteotomy [5, 19, 31]. The mean reduction in posterior tibial slope was 3° (95% CI, 2.2 to 3.8) (one study reported *p* < 0.01; in two studies, *p* value was NR). One study (*n =* 1) reporting posterior tibial slope indicated the use of an opening-wedge osteotomy [29]. The mean reduction in posterior tibial slope was 2° (*p* value was NR). One study with 36 patients reported a positive correlation between KT-1000 side-to-side difference and posterior tibial slope (*p* < 0.05; *r* = 0.6) (Table 2) [31].

#### Objective International Knee Documentation Committee Scores

Global International Knee Documentation Committee (IKDC) was reported in four studies (*n =* 47) [5, 19, 23, 31]. Overall, pre-operatively, 54.3% of patients were graded D (severely abnormal function), 43.3% were graded C (abnormal function), and 2.2% were graded B (nearly normal function), while post-operatively, 53.2% were graded B (nearly normal function), 36.2% were graded A (normal function), 4.3% were graded C, and 6.4% were graded D (Table 2) [5, 19, 23, 31].

#### Osteoarthritis

Osteoarthritis was examined in four studies (*n =* 60) [5, 29–31]. In these four studies, 51.6% (*n =* 31) of the patients demonstrated radiographic signs of osteoarthritis (two studies reported medial compartment, and two studies did not report compartment) before ACLR revision, and 58.3% (*n =* 35) of the patients demonstrated radiographic signs of osteoarthritis at final follow-up after ACLR revision.

### Clinical Subjective Outcomes

This review reported several clinical subjective outcomes. The greatest improvements were seen in subjective IKDC scores (*n =* 63) (two studies reported *p* < 0.05; in four studies, *p* value was NR), Tegner–Lysholm scores (*n =* 28) (one study reported *p* < 0.05; in three studies, *p* value was NR), and pain scores on a visual analog scale (VAS) (*n =* 50) (in three studies, *p* value was NR). Meanwhile, Tegner activity scale scores showed the least improvement (one study reported *p* < 0.05; in two studies, *p* value was NR). Three studies reported return-to-sport (*n =* 7) at follow-up (3 to 78 months) [19, 23, 29]. The return-to-sport rate was 85.7% (*n =* 6). Quality of life (*n =* 47) based on EQ-5D scores and subjective patient satisfaction were excellent. Study-specific data has been outlined in Table 3 [1, 5, 19, 23, 29–31].

**Table 3:** Clinical subjective outcomes

Author	Subjective IKDC	Tegner–Lysholm	Tegner activity	Return to sports	Pain	Quality of life/patient satisfaction	Complication
Dejour et al. [5]	44.1 ± 16.1 (pre-op) to 71.6 ± 6.15 (median, 72.8; range, 62.2–78.5) (post-op)	38.4 ± 16.4 (pre-op) 73.8 ± 5.8 (median, 74; range, 65–82) (post-op)	NR	NR	NR	4 excellent, 3 good, 2 fair	NR
Zaffagnini et al. (2013) [31]	58.0122 (pre-op) to 72.0165 (post-op)	NR	3 (range 2–4) (pre-op) to 5 (range 4–5) (post-op)	NR	VAS: 73.2120 (pre-op) to 42.1250 (post-op)	EQ-5D from 0.62023 (pre-op) to 0.89013 (post-op)	NR
Zaffagnini et al. (2012) [30]	55.33 ± 12.63 (pre-op) to 71.27 ± 16.26 (post-op)	NR	4.0 (IQR 2.4) (pre-op) to 4.0 (IQR 4.5)	NR	7.710 ± 1.250 (pre-op) to 4.710 ± 2.870	NR	1 case of stiffness
Baverel et al. [1]	45.3 (pre-op) to 76.5 (post-op)	40.8 (pre-op) to 75.6 (post-op)	NR	NR	NR	NR	NR
Sonnery-Cottet et al. [19]	39.5 (range, 21.8–64.4) (pre-op) to 79.1 (range, 48.3–98.9) (post-op)	46.2 (range, 26–69) (pre-op) to 87.8 (range, 60–100) (post-op)	7.4 (pre-op) to 7.2 (post-op)	All but 1 returned to sport at same activity level; 2 patients with 13.14° posterior tibial slopes returned to sport at the same level	NR	NR	NR
Walker et al. [23]	73.6 (post-op)	90 (post-op)	NR	Patient returned to biking, elliptical work, and a job with 12 h of walking and standing	VAS 0	NR	NR
Yonekura et al. [29]	NR	NR	NR	Returned to mountain climbing	NR	Satisfied	NR

*NR* not reported, *IQR* interquartile range, *VAS* visual analog scale, *IKDC* International Knee Documentation Committee

### Complications

Only one complication of stiffness was reported, for which arthroscopic arthrolysis was performed. There were no reported ACL re-ruptures or subsequent revision surgeries.

## Discussion

The most important finding in this systematic review was that revision ACLR combined with HTO resulted in significant improvements in anterior tibial translation, anterior laxity, posterior tibial slopes (especially in patients with posterior tibial slopes greater than 12°), subjective IKDC scores, Tegner–Lysholm scores, and VAS pain scores. The study also found few reported complications. Notably, there was a 0% re-rupture rate compared to ACLR revision failures rates ranging from 2 to 28% [7]. Individuals with increased posterior tibial slopes are known to have higher ACL rupture rates [15, 24]. In fact, patients with a tibial slope greater than 12° have been reported to have increased odds (by a factor of 5) to incur future ACL injury [24]. Thus, the theoretical ability of HTO to lower re-rupture rates through a reduction in posterior tibial slope is promising in revision settings [15, 24]. However, this re-rupture rate may be a gross underestimate due to a small sample size, limited follow-up time, and selective reporting.

Objective measurements show this procedure was successful in resolving anterior instability. It has been previously contended that HTOs, especially in ACL-deficient patients or other primary settings, may aid in controlling anterior laxity by correcting sagittal imbalance [13]. This corresponds with findings from one of the included studies, which demonstrated a positive correlation between posterior tibial slope and KT-1000 side-to-side differences in anterior laxity [31]. Based on such findings, HTOs can be used with ACLR revision to help control excessive anterior laxity.

This systematic review found radiographic signs of osteoarthritis pre-operatively in 51.6% (*n =* 31) and post-operatively in 58.3% (*n =* 35) of the 60 patients examined for osteoarthritis. In a case series (*n =* 107) that studied solely ACLR revision patients, 80.7% of patients went on to have osteoarthritis [6]. Considering that HTO may make future total knee replacements more difficult, it should be used cautiously; the results of this study suggest that a large number of these patients will still go on to develop knee osteoarthritis [18]. Still, no available data showed what percentage of these patients requires conversion to total knee arthroplasty and what percentage of patients with radiographic evidence of osteoarthritis was symptomatic. Furthermore, although a high percentage of patients went on to develop osteoarthritis despite combined HTO and ACLR, the osteoarthritis may have been more severe with the combined procedure. Unfortunately, there is no data available to confirm this hypothesis. It should be noted that the mean age of patients in this study was 38 years, and in the revision setting, patients may have been predisposed to osteoarthritis from other injuries (i.e., to cartilage or meniscus) at the index procedure.

Only one complication was reported in the included studies; we suspect that complications may have been underreported in studies included in this systematic review, possibly due to poor follow-up or documentation methods. In one study exploring primary ACLR with HTO, complications such as deep venous thromboses, intra-articular fractures, and peroneal nerve injuries were all reported [9]. Such complications may be at least as common, if not more so, in the revision setting. Furthermore, the addition of HTO to ACLR adds a subset of complications to the procedure, such as failure of fixation, loss of correction, non-union or delayed union, and patella baja [11, 28]. Finally, adding HTO to an already technically challenging ACLR revision may lead to increased technical errors by surgeons.

The main indications found for combined HTO with ACLR revision were a posterior slope greater than or equal to 12° and severe varus malalignment [5]. Using these indications, the studies observed improvements in various knee stability and function measures, including a clinically significant reduction in pain scores, improvement in subjective and objective IKDC scores, and reasonable return-to-sport rates. Included studies noted that the HTO was successful in reducing the posterior slope of the tibia in patients with increased posterior tibial slope.

The strength of this systematic review stems from the rigorous methodology used; multiple databases, a broad search strategy, and a duplicate systematic approach to reviewing the literature ensured that no relevant articles were overlooked. The screening process did not have exclusion criteria against non-English studies or gray literature, thereby minimizing publication bias. The systematic screening approach was employed in duplicate, thus minimizing reviewer bias. Excellent agreement at all screening stages and quality assessment were obtained.

The most significant limitations of this review are the lack of high-quality studies with large sample sizes in the literature pertaining to simultaneous HTO and ACLR revision and the heterogeneity (due to inconsistency in patients, pathology, surgical techniques, length of follow-up, and outcomes). All studies in this review are of level III and IV evidence, with small sample sizes. The poor documentation of data (e.g., mean follow-up time, demographics, reasons for previous failure outcomes such as coronal alignment, and complications) across included studies may be attributed to low-quality evidence with poor follow-up methods or documentation of data. Ultimately, this limits our ability to make definitive conclusions.

Future studies with long-term follow-up, larger sample sizes, and prospective design are needed to further confirm the results found in existing studies. Ideally, future studies will have improved documentation of necessary data (i.e., demographics, outcomes, graft failures, and complications), which is often lacking in the available literature. Currently, there is no comparative data on patients who receive a combined HTO and ACLR versus patients who receive ACLR alone or HTO alone; therefore, although the results were overall positive in this review, it is unclear how much the addition of the HTO adds to these outcomes. Finally, future studies should determine a threshold/cutoff for when osteotomies should be performed in ACLR revision.

The use of HTO along with ACLR revision seems promising due to the good post-operative functional outcomes, low complication rates, and no observed re-ruptures. The main indications for combined HTO in ACLR revision were a posterior slope greater than 12° or severe varus malalignment. However, future studies with large sample sizes and long-term follow-up are required to confirm these preliminary results.

### Electronic Supplementary Material

ESM 1 (PDF 3079 kb)

## Appendix

**Table 4:** Search strategy across online databases

Medline	Embase	PubMed
Strategy	Strategy	Strategy
1. exp anterior cruciate ligament reconstruction/or exp anterior cruciate ligament/or ACL.mp.	1. exp anterior cruciate ligament reconstruction/or exp anterior cruciate ligament/or ACL.mp.	((“2017/2/27”[Date - Publication]: “3000”[Date - Publication])) AND (((((ACL) OR Anterior Cruciate Ligament Reconstruction [MESH]) OR Anterior cruciate ligament [MESH])) AND (((slope) OR revision) OR Osteotomy))
2. exp. osteotomy/or osteotomy.mp.	2. exp. osteotomy/or osteotomy.mp.	
3. revision.mp.	3. revision.mp.	
4. slope.mp.	4. slope.mp.	
5.2 or 3 or 4	5.2 or 3 or 4	
6.1 and 5	6.1 and 5	
7. Limit 6 to human	7. Limit 6 to human	
Retrieved: 1095 studies	Retrieved: 1616 studies	Retrieved: 247 studies
